# A Qualitative Study to Identify Perceptual Barriers to Antiretroviral Therapy (ART) Uptake and Adherence in HIV Positive People from UK Black African and Caribbean Communities

**DOI:** 10.1007/s10461-019-02670-x

**Published:** 2019-09-13

**Authors:** Elizabeth Glendinning, Johanna Spiers, Jonathan A. Smith, Jane Anderson, Lucy J. Campbell, Vanessa Cooper, Rob Horne, the SUPA Group, Jane Anderson, Lucy J. Campbell, Trudie Chalder, Simon Collins, Vanessa Cooper, Elizabeth Glendinning, Scott Harfield, Rob Horne, Kathryn King, Heather Leake Date, Paul McCrone, Iris Mosweu, Susan Michie, Mark Nelson, Nicky Perry, Caroline Sabin, Jonathan A. Smith, Winnie Sseruma, Sarah Walker

**Affiliations:** 1 Centre for Behavioural Medicine, UCL School of Pharmacy, BMA House, Tavistock Square, London, WC1H 9JP UK; 2 Department of Psychological Sciences, Birkbeck, University of London, London, UK; 3 Centre for the Study of Sexual Health and HIV, Homerton University Hospital, London, UK; 4 HIV Research Centre, King’s College London, London, UK

**Keywords:** HIV, Antiretroviral, Beliefs, Necessity, Concerns, Adherence

## Abstract

To inform the development of interventions to increase uptake and adherence to antiretroviral therapy (ART), we explored perceptions of ART in semi-structured interviews with 52 men and women from UK black African and black Caribbean communities. Verbatim transcripts were analyzed using framework analysis. Perceptions of ART could be grouped into two categories: doubts about the personal necessity for ART and concerns about potential adverse effects. Doubts about necessity stemmed from feeling well, doubts about the efficacy of ART, religious beliefs and the belief that treatment was futile because it could not cure HIV. Concerns about adverse effects included the fear that attending HIV services and taking treatment would lead to disclosure of HIV, feeling overwhelmed at the prospect of starting treatment soon after diagnosis, fears about side effects and potential long-term effects, and physical repulsion. The findings will facilitate the development of interventions to increase uptake and adherence to ART.

## Introduction

Effective antiretroviral therapy (ART) has dramatically reduced morbidity and mortality associated with HIV infection [1] and can prevent transmission to sexual partners [2]. Good adherence to effective treatment should not only improve physical health, but also psychological wellbeing by empowering patients to take an active role in managing their condition [3]. Modelling studies have shown that if sufficient numbers of people living with HIV (PLWH) are diagnosed and take sustained and effective treatment, new HIV infections could be eradicated within the next two decades [4]. In 2014, the Joint United Nations Programme on HIV/AIDS (UNAIDS) proposed a target of ending the AIDS epidemic by 2030 [4]. The report set out three milestones that would need to be achieved by 2020 in order to reach this target: (1) 90% of all people with HIV knowing their HIV status; (2) 90% PLWH who know their diagnosis receiving continuous treatment and (3) 90% of people with diagnosed HIV having viral suppression [4].

At the end of 2017, globally 75% of PLWH knew their status, 79% of those who knew their diagnosis were on treatment and 81% of those on treatment had viral suppression [5]. In the UK, the UNAIDS targets had been met [6, 7]. Despite these successes, delay to treatment uptake and poor adherence to medication continue to pose important challenges. The number of people with diagnosed HIV who were not taking ART was likely to be an underestimate because not everyone who is prescribed ART actually receives or takes it [8]. Furthermore, although nonadherence is a common cause of treatment failure, it is not synonymous with undetectable viral load. Several studies show that nonadherence to ART remains suboptimal: a meta-analysis of 84 studies across 20 different countries found the mean rate of ART adherence (defined as ≥ 90%) was 62% [9]. Since rates of adherence tend to decline over time [10], it is important that barriers to adherence are addressed before viral load becomes detectable.

There is a comprehensive literature on factors influencing adherence to ART. In common with most behaviors, adherence is a product of motivation and ability [11]. In order to optimize ART uptake and adherence, there is a need to understand why people with HIV may not want or be able to initiate and continue to take ART. One approach is to consider the perceptions (e.g. beliefs about HIV and ART) that may influence motivation together with the practicalities (e.g. capability and resources) influencing the ability to adhere.

The beliefs influencing uptake and adherence can be conceptualized by the Necessity Concerns Framework (NCF). Studies across the UK and the USA show that beliefs about personal necessity for ART and concerns about potential adverse effects predict delay to uptake and nonadherence [10, 12–15]. These studies used the Beliefs about Medicines Questionnaire (BMQ), a valid and reliable method of quantifying adherence related beliefs (Necessity Beliefs and Concerns) using items identified in qualitative studies [16, 17]. While previous studies have found that the NCF is relevant in minority ethnic groups [13] it is not clear whether the items in the BMQ capture all of the salient issues in people from black African and black Caribbean communities in the UK. Lower rates of engagement with treatment and care have been observed within these communities when compared to the overall UK population of people living with HIV [18, 19].

Factors influencing engagement with HIV care have previously been identified in studies conducted in Africa and the Caribbean, as well as with people from black African communities living in the UK. A recent systematic review (n = 59) of studies reporting on factors influencing decision-making among PLWH in Africa identified three groups of challenges: (1) those associated with poverty, stigma and unpredictable life events, (2) challenges relating to the health care system, and (3) self-efficacy and social, financial and practical support [20]. A systematic review of studies conducted in Latin America and the Caribbean (n = 53) identified several barriers to adherence to ART among PLWH in the Caribbean [21], including perceptions of HIV providers, alcohol use, a lack of social support for adherence, having children and experiences of side effects [21].

Studies conducted with people from black African communities living in the UK have identified several barriers to accessing healthcare services including HIV-associated stigma, fear of discrimination, the perception that accessing healthcare was unnecessary in the absence of symptoms and competing priorities (such as housing and employment issues) [22]. Fewer studies have identified barriers to ART uptake and adherence. A study conducted 20 years ago identified several concerns about ART among black African PLWH living in London including concerns about short and long-term side effects, lack of confidence in ART, distrust of the medical profession and fears of discrimination [23]. One study found lower attendance in care among black African PLWH who believed that only God can cure HIV; however, religiousness (e.g. attendance at religious services and religious beliefs) was not associated with initiation of ART or subsequent changes in viral load [24]. The quality of doctor-patient relationships may be an important determinant of adherence to ART: in a study of African migrants in London, some participants felt that their concerns about side effects had not been taken seriously, and had stopped treatment as a result [25].

Given the lack of current research focusing on perceptual barriers to ART uptake and adherence among black African and black Caribbean PLWH in the UK, the aim of this study was to identify the salient perceptual barriers to ART uptake and adherence in a sample of people of black African and black Caribbean descent living with HIV in London.

## Methods

This was a cross-sectional, qualitative, interview-based study. Qualitative methodologies are optimal to examine participants’ views and experiences [26].

### Sampling

Study participants were recruited from specialist HIV outpatient clinics at Homerton University Hospital and King’s College Hospital in London, UK. Patients were eligible if they were ≥ 18 years, had a diagnosis of HIV, were of black African or Caribbean ethnicity, were born in Africa or the Caribbean, had been prescribed ART for ≥ 12 months and were assessed by clinicians as being nonadherent to ART according to 2012 British HIV Association (BHIVA) guidelines [27]. Purposive sampling was used to reflect a broad range of geographical provenance and stage of HIV infection (asymptomatic vs symptomatic) (Table 1).

**Table 1:** Sample characteristics (n = 52)

Demographic or clinical characteristic		
Gender		
Female	n (%)	35 (60)
Age		
Years	Median (IQR)	44 (37–49)
Region of birth		
West Africa	n (%)	23 (44)
East Africa	n (%)	17 (33)
Other Africa	n (%)	2 (4)
Caribbean	n (%)	10 (19)
Employment		
Professional	n (%)	8 (15)
Manual	n (%)	5 (10)
Unemployed	n (%)	37 (71)
Student	n (%)	2 (4)
Symptomatic status^a^		
Symptomatic	n (%)	28 (53)
Asymptomatic	n (%)	24 (46)
Years since HIV diagnosis	Median (IQR)	9 (3–12)
Years since treatment start	Median (IQR)	6 (3–9)

^a^Symptomatic status was determined with the US Centers for Disease Control and Prevention (CDC) classification system: Stage A: free from clinical symptoms such as opportunistic infections; CDC stage B or C: experiencing symptomatic conditions that are attributed to HIV, indicate a defect in cell mediated immunity are complicated by HIV infection or the presence of an AIDS-indicator condition

### Recruitment

Potential participants were informed about the study by their HIV clinicians and introduced to study researchers during routine visits. Consistent with the main study aim, patients were told that the purpose of the study was to understand their perceptions and experiences of ART. Written informed consent was obtained. Approval for this study was granted by the City and East London Research Ethics Committee (11/LO/0970). Participants were reimbursed £20 for their time and expenses.

### Data Collection

Data were collected through in-depth, individual face-to-face interviews conducted in clinical settings by experienced qualitative researchers (EG, JSp). Interview guides were semi-structured, consisting of open-ended questions and prompts to explore participants’ experiences of ART, including what taking treatment meant to the participant and its importance in their life, how they took treatment, and whether they had concerns about taking ART (and conversely not taking it). Demographic characteristics were obtained by a self-report questionnaire.

### Data Analysis

An independent service transcribed each recording verbatim, which was then reviewed for quality by the Researcher who conducted the interview (EG or JSp). Data were analyzed using framework analysis [28]. This method was chosen because it allows for the inductive exploration of patients’ accounts while also enabling the exploration of pre-defined theory [28]. Our analysis was guided by the Necessity Concerns Framework [16]. Each transcript was read whilst listening to the recording. Two researchers (EG and JSp) initially developed a framework of key issues and themes. This framework was used to code a further subset of interviews and was refined through discussion. A comprehensive data chart was then constructed by extracting segments of data from the transcripts and arranging these in a matrix according to emerging themes. The corpus of extracts was managed using NVivo 10.0.

## Results

Fifty-two patients of West African (n = 23), East African (n = 17), Central/South African (n = 2) and Caribbean (n = 10) heritage were interviewed between February and August 2012. Interviews were conducted in English (50) and French [2]. Ten themes were identified. These themes could be grouped under two main categories: five themes were categorized as doubts about the necessity for treatment and five were categorized as concerns about adverse effects (Table 2).

**Table 2:** Perceptions of ART identified in framework analysis

Doubts about the necessity for ART
No symptoms, no problem
Fatalistic beliefs
Beliefs linked to stigma and shame
Conflict regarding the roles of God and medicine
Doubts about effectiveness
Concerns about ART
Insufficient time to address concerns
Risk of disclosure of HIV through ART
Decreased quality of life from side effects
Physical repulsion
Concerns about long-term effects

### Doubts About the Necessity for ART

#### No Symptoms, No Problem

The rationale for taking ART was sometimes unclear to people who were not experiencing specific symptoms that they associated with HIV. These participants described taking treatment as being at odds with their idea of the purpose of medication, which was to alleviate symptoms, rather than as a strategy to prevent them from becoming ill. Many people found it difficult to accept the need for ART when they were feeling fine.

If you have a headache you say let me take paracetamol to make [it] stop. And you’re told you have HIV, but you don’t have symptoms, you are not sick from it and you are told “take medication”. I don’t have headache and they are giving me medication to stop the headache. (Female (F), 40, Zimbabwean, symptomatic, 16 years since diagnosis).

#### Fatalistic Beliefs About HIV

Many participants initially believed that they would die from HIV. For some, this belief was based on previous experiences, such as experiencing a family member’s death from AIDS-related illness. Some participants felt that because HIV is incurable, they would die from it, and therefore there was no point to taking medication. Taking medicine each day to control HIV did not fit with these participants’ perception of HIV as a fatal illness.

It’s an incurable disease, you’re going to die. It’s like having a death sentence hanging over you and the shock, the despair… it does affect the taking of the medication because you think at the end of the day what’s the use, what’s the point of using it, because you’re going to die. It’s not curable. (M, 47, Zambian, symptomatic, 10 years since diagnosis)

#### Beliefs Linked to Stigma and Shame

Participants frequently described having experienced feelings of shame and self-blame after being diagnosed HIV positive. For some participants, the sense of shame felt about being HIV-positive was so great that they did not want to take treatment to live.

HIV is a punishment… So I cannot live my life… I thought maybe, I was hoping, I was going to die quickly. (M, 54, Zambian, symptomatic, 12 years since diagnosis)

Others did not accept that their diagnosis of HIV was correct, considering that the diagnosis must have been made in error or attributing symptoms or test results to another condition. In this context, it did not make sense to participants to take ART.

I am still having doubt [about the diagnosis] until it is proven right…. If it’s a mistake somewhere I think the earlier we can identify it the better. Something else can be wrong with my blood… It might be something new or modern. (Male (M), 45, Ivorian, symptomatic, 2 years since diagnosis)

#### Conflict Regarding the Roles of God and Medicine

Some participants had a strong conviction that HIV could be cured by their belief in God. This was strengthened by witnessing testimonies in church from parishioners who claimed that they were no longer HIV positive following sufficient prayer. A participant recounted that a parishioner showed a letter from their doctor stating that they were cured of their HIV. For these participants, being healed was a measure of one’s strength as a believer. Remaining HIV positive was interpreted by some as an indication of inadequate belief in God on their part to be able to receive a cure.

You might see someone who has had the same sickness for 40 years, and he still hasn’t been healed, and then you will see someone who has just suffered 2 years and miraculously he will be healed. It’s all about faith. That is what I believe, that it all depends on faith. (M, 47, Zambian, symptomatic, 11 years since diagnosis)

For some participants, there was an internal conflict regarding the role of God and ART in the treatment of HIV. Although they expressed the resolute belief that only faith would prevent them from becoming ill, this was inconsistent with the fact that they were taking at least some of their treatment.

#### Doubts About Effectiveness

Doubts about the effectiveness of ART stemmed from participants’ common-sense beliefs about their medicines, which were often at odds with the medical rationale for treatment. For example, some participants perceived that ART would not be necessary if they were able to boost their immune system in a more organic way, e.g. by eating healthily or taking natural remedies. Some participants had misconceptions about their treatment, for example the belief that efficacy was associated with the number of tablets they were prescribed. This is illustrated by the following quote, where the participant was concerned that her current one-tablet regimen would be less effective than the 4-tablet regimen she had taken previously:

This new one you only take it once a day, are they reducing it down because I’m taking [ART for] too long? I wonder if I’m going to [be] dead soon because they’re reducing it down (F, 38, Ugandan, asymptomatic, 2 years since diagnosis)

### Concerns About ART

#### Insufficient Time to Come to Terms with an HIV Diagnosis Before Starting ART

The majority of participants had been diagnosed with HIV at a late stage of infection and had been recommended to start ART immediately. Many participants were devastated and overwhelmed by their diagnosis and found it difficult to both come to terms with being HIV-positive and commit to life-long treatment at the same time. In this context, participants described having difficulty taking in the information they were given. There was insufficient time for them to articulate their concerns about ART to their doctor, meaning they started treatment with strong concerns about taking it.

At first they will just be talking to you and everything is just going in other ways. You’ll just be sitting and they are talking to you but when you leave they’ll know everything’s gone. Yes, just passing through into your ears and out on top of your head. (F, 41, Ugandan, symptomatic, 2 years since diagnosis)

#### Risk of Disclosure of HIV Through ART

Many people explained that if other people in their communities became aware of their HIV status, they risked severe social and economic consequences. This was particularly true for people who were dependent on small networks of support, those who faced immigration restrictions, or who were staying with friends or acquaintances and who faced the risk of ostracism or homelessness if people knew that they were living with HIV. Some participants had concerns about attending the HIV clinic, in case they recognized members of their community, or taking treatment, in case the physical characteristics of the medication, such as large or colorful tablets attracted attention, making their HIV status visible.

I don’t have a house, so I was sleeping with friends… I can’t take the medication because maybe they will start talking about me and maybe they would refuse me sleeping in their house. (F, 37, Nigerian, asymptomatic, 2 years since diagnosis)

#### Decreased Quality of Life from Side Effects

Many participants experienced side effects from their ART and associated taking treatment with feeling ill. Taking ART was perceived by some to have reduced rather than increased their quality of life. This was especially true for participants who had not been experiencing symptoms that they associated with HIV before initiating treatment. For some, the experience of taking ART was so adverse that the promise of longer-term health was not enough to outweigh the negative experience of taking medication.

I don’t see what they’re doing for me, all they’re doing they’re just making me feel worse. (F, 35, Jamaican, asymptomatic, 4 years since diagnosis)

#### Physical Repulsion

Many patients talked about the physical attributes of the tablets themselves. Some reported difficulty in swallowing, while others found the taste and size of the tablets off-putting or nausea-inducing. These barriers led participants to view ART as an impediment in their lives and at times even a source of repulsion.

I just want to throw up. Sometimes I throw up and think, good, it’s out of my body now. (M, 32, Guyanese, 4 years since diagnosis)

#### Concerns About Long Term Effects

Concerns about long-term effects of ART were common. Many patients feared that ART was toxic and were convinced it would cause damage to their body in the long-term, a situation which they perceived as no better than letting HIV take its course. For some patients, negative experiences of ART such as side effects tipped the balance in favor of nonadherence as the preferable health strategy.

I was saying you are trying to get me better but at the same time you’re killing me. (F, 52, Jamaican, asymptomatic, 3 years since diagnosis).

## Discussion

This study identified perceptual barriers to ART in people of African and Caribbean heritage living in the UK. Consistent with studies conducted across long-term conditions [29], these barriers could be grouped into two categories—doubts about the necessity for ART and concerns about adverse effects. The findings are consistent with an extended common-sense model of self-regulation [11], which proposes that although the behaviors that patients adopt to cope with their illness are often at odds with medical advice, they make sense when viewed in the light of patients’ beliefs about their illness and treatment. Understanding patients’ common-sense perspectives of their condition and treatment is an important first step in the development of strategies to increase adherence.

Five of the beliefs identified represented doubts about the necessity for ART. The perception that treatment is not necessary in the absence of symptoms and a preference for non-pharmacological methods of controlling HIV were consistent with those identified in a previous study conducted with men who have sex with men (MSM) who declined a clinically indicated recommendation to initiate ART [17]. The belief that faith in God can cure HIV was previously identified in a study of newly diagnosed black African men and women receiving treatment for HIV in London, where over a third of frequent church attenders believed that HIV could be cured by faith in God [24]. Other beliefs identified in the current study which may influence patients’ perceptions of their personal necessity for ART include patients’ doubts about the validity of their HIV diagnosis and a fatalistic view of HIV as an incurable, untreatable disease.

Five of the themes identified in this study related to patients’ concerns about ART. In common with previous studies [10, 14, 17], many patients had concerns about side effects, potential long-term effects due to toxicity and a physical repulsion to ART. Fears of discrimination leading to the need to conceal one’s HIV status from others have been previously documented among people from black African communities in Europe [30–32], and HIV stigma has been associated with lower adherence [33]. An additional concern identified in this study was a lack of time between the diagnosis of HIV and the recommendation to initiate ART. Many people reported being overwhelmed by their simultaneous diagnosis of HIV and committing to life-long treatment. In this context, patients felt that they did not have sufficient time to articulate their concerns about ART before initiating treatment. This is an important consideration given that late diagnosis (CD4 count < 350 cells/mm^3^) is common among people from black African and black Caribbean communities in the UK [27, 34] meaning that a significant proportion of people in these populations are recommended immediate initiation of ART. Importantly, UK and international HIV treatment guidelines now recommend that patients should start ART as soon as possible after diagnosis, regardless of their CD4 count, so prescribing practices will need to consider the patient’s preparedness to ensure a successful treatment initiation [1]. Evidence that people who have strong concerns about ART at the time of a treatment recommendation are more likely to decline treatment or to be nonadherent 12 months later highlights the need to elicit and address concerns about ART at this stage of the care pathway [10]. PLWH in this study described being unable to take in or recall the information that they were given in this context, demonstrating the need for tailored interventions and support with appropriate information resources.

These findings show the importance of understanding patients’ beliefs about HIV and ART in order to ensure that the rationale for ART can be communicated in a way that makes sense to the individual and which does not conflict with their existing beliefs. Most of the themes identified in this study are present in the Beliefs about Medicines Questionnaire (BMQ-ART) [10, 16], which can be used to elicit beliefs about ART. Cognitive behavioral techniques may be useful to explore and challenge unhelpful or inaccurate beliefs. Patients’ doubts about their personal necessity for ART may be addressed by presenting the medical rationale for ART in a way that addresses or aligns with a patient’s common sense view. Concerns about side effects may be addressed during consultations by routinely discussing unwanted effects that they may be experiencing, providing relevant pharmaceutical treatment and advice that is tailored and appropriate to the individual. Fears about potential unwanted and adverse effects may be addressed by exploring the potential benefits of ART with the patient and weighing this up against the likelihood of short and long-term unwanted effects. In this study, many patients were concerned that their HIV status would be revealed to others by the conspicuously large size and vivid color of their ART tablets. It is possible that generic medicines, which are less distinguishable in their appearance, may help address these concerns.

The findings of this study should be interpreted in the light of its limitations. This was a cross sectional study and although patients were asked about different points in their treatment journey, they were not followed over time, therefore we were unable to determine which beliefs were more prevalent at each timepoint (diagnosis, recommendation of treatment and ongoing adherence). While it is possible that some patients may not have been forthcoming in disclosing nonadherence, the range of beliefs elicited suggests that patients felt able to discuss the issues pertinent to them. The use of qualitative methods was appropriate to examine beliefs about ART in previously under-researched groups, however it was not possible to determine the strength the beliefs identified using this approach. Further studies using quantitative methods are required to determine how strongly these beliefs are held and whether they differ between cultural groups. Since the interviews were conducted there have been significant developments in research and treatment which may impact on patients’ beliefs about ART and treatment decisions. Following robust evidence that individuals with an undetectable viral load on ART are not able to transmit HIV to others [2] a test and treat approach where all people diagnosed with HIV are recommended to start ART as soon as possible has been adopted [35]. This has led to initiatives to help individuals to initiate ART as close as possible to receiving their diagnosis. Furthermore, since these interviews were conducted there has been the development of newer fixed dose, single tablet regimens, and generic medicines consisting of smaller, more discreet pills tablets which may reduce concerns about disclosure of HIV through ART.

## Conclusions

In order to increase uptake and adherence to ART, the National Institute for Clinical Excellence (NICE) recommends eliciting and addressing both perceptual and practical barriers to treatment. This study has identified perceptual barriers to ART in previously under-researched UK populations and may therefore be useful in clinical practice and for those developing evidence-based interventions to support uptake and adherence to ART. The findings will inform the development of an intervention to increase individual motivation to initiate ART and maintain a high level of adherence over the long-term, in order to optimize physical and psychological outcomes for people living with HIV and help to achieve and maintain the targets set by UNAIDS.
